# The evolution of health policy guidelines for assisted reproduction in the Republic of Ireland, 2004-2009

**DOI:** 10.1186/1478-4505-9-28

**Published:** 2011-06-24

**Authors:** David J Walsh, Mary L Ma, Eric Scott Sills

**Affiliations:** 1 Division of Reproductive Endocrinology & Infertility, The Sims Institute-Sims IVF/Department of Obstetrics & Gynaecology, School of Medicine, Royal College of Surgeons in Ireland; Dublin, Ireland

## Abstract

This analysis reports on Irish regulatory policies for in vitro fertilisation (IVF) from 2004-2009, in the context of membership changes within the Medical Council of Ireland. To achieve this, the current (2009) edition of the *Guide to Professional Conduct & Ethics *was compared with the immediately preceding version (2004). The statutory composition of the Medical Council from 2004-2009 was also studied. Content analysis of the two editions identified the following differences: 1) The 2004 guide states that IVF "should only be used after thorough investigation has failed to reveal a treatable cause of the infertility", while the 2009 guide indicates IVF "should only be used after thorough investigation has shown that no other treatment is likely to be effective"; 2) The 2004 stipulation stating that fertilized ovum (embryo) "must be used for normal implantation and must not be deliberately destroyed" is absent from the 2009 guidelines; 3) The option to donate "unused fertilised ova" (embryos) is omitted from the 2009 guidelines; 4) The 2009 guidelines state that ART should be offered only by "suitably qualified professionals, in appropriate facilities, and according to the international best practice"; 5) The 2009 guidelines introduce criteria that donations as part of a donor programme should be "altruistic and non-commercial". These last two points represent original regulatory efforts not appearing in the 2004 edition. The Medical Practitioners Act 2007 reduced the number of physicians on the Medical Council to 6 (of 25) members. The ethical guidelines from 2004 preceded this change, while the reconstituted Medical Council published the 2009 version. Between 2004 and 2009, substantial modifications in reproductive health policy were incorporated into the Medical Council's ethical guidelines. The absence of controlling Irish legislation means that patients and IVF providers in Ireland must rely upon these guidelines by default. Our critique traces the evolution of public policy on IVF during a time when the membership of the Medical Council changed radically; reduced physician contribution to decision-making was associated with diminished protection for IVF-derived embryos in Ireland. Considerable uncertainty on IVF practice in Ireland remains.

## Introduction

At present, there is no legislation specifically regulating assisted reproductive technology (ART) in the Republic of Ireland [1]. While the need for a statutory framework to address the provision of ART in Ireland was acknowledged by the Commission on Assisted Human Reproduction (CAHR) in 2005 [2]  as well as the Irish judiciary in 2009 [3], the Parliament of the Republic of Ireland (the Oireachtas) has yet to enact any relevant statutes.

In the absence of controlling legislation addressing IVF, the only guidance medical practitioners in Ireland currently have on assisted fertility matters is provided by *The Guide to Professional Conduct & Ethics for Registered Medical Practitioners *(the "ethical guide"). The version currently in effect is the most recent edition published in 2009 [4], while the immediately preceding edition appeared in 2004 [5]. These guides are periodically issued by the Medical Council, which is the central medical registration authority in Ireland. With a remit covering all medical practitioners throughout Ireland and not just those engaged in the practice of reproductive medicine, the council's guidelines must provide guidance across the full range of medical practice. In this report we examine this document in detail, with an emphasis on how the council's position on ART has evolved between 2004 and 2009, on how the council incorporated recommendations from the CAHR into its 2009 guidelines, and how the council's own membership changed between 2004 and 2009.

## Discussion

### Medical Council ethical guidelines 2004 and 2009: Content analysis

The sixth (2004) edition of the Medical Council's ethical guidelines allocated 162 words to the area of ART, touching upon cryopreservation, donation, and IVF [5]. In contrast, the seventh (2009) edition devoted 149 words to the topic of assisted fertility [4]. At least four concepts central to the advanced reproductive technologies found different expression in these guidelines between 2004 and 2009 (see Table 1).

**Table 1:** Comparison of medical practice guideline sections related to assisted fertility treatments in the Republic of Ireland, as issued by the Commission for Assisted Human Reproduction and the Medical Council

Policy issue	**6**^**th **^**Edition ethical guide**^**a**^	**7**^**th **^**Edition ethical guide**^**b**^	**CAHR**^**c**^
			

**Agency, accreditation, and data tracking**	[*none*]	20.2 Assisted reproduction services should only be provided by suitably qualified professionals, in appropriate facilities, and according to international best practice. Regular clinical audit and follow-up of outcomes should be the norm.	1. A regulatory body should be established by an Act of the Oireachtas to regulate AHR services in Ireland. 2. National statistics on the outcome of AHR techniques in Ireland should be compiled and made available to the public. 3. Longitudinal studies of children born as a result of AHR should be established, in accordance with standard ethical/legal requirements and with the consent of families, in order to facilitate long-term monitoring.

**Gamete donation**	24.4 [...] Doctors who consider assisting with donation to a third party must have regard to the biological difficulties involved, and pay meticulous attention to the source of the donated material. 24.5 [...] If couples have validly decided they do not wish to make use of their own fertilised ova, the potential for voluntary donation to other recipients may be considered.	20.3 If you offer donor programmes to patients, you must consider the biological difficulties involved and pay particular attention to the source of the donated material. Such donations should be altruistic and non-commercial. You should keep accurate records for future reference.	10. Appropriate guidelines should be put in place by the regulatory body to govern the options available for excess frozen embryos. These would include voluntary donation of excess healthy embryos to other recipients, voluntary donation for research or allowing them to perish. 19. Donation of sperm, ova and embryos should be permitted and should be subject to regulation by the regulatory body. 21. Appropriate guidelines should be put in place to govern the selection of donors; to screen for genetic disorders and infectious disease; to set age limits for donors and to set an appropriate limit on the number of children to be born by the use of sperm or ova from a single donor. 23. Donors should not be paid nor should recipients be charged for donations per se. This does not preclude payment of reasonable expenses and payment for AHR services.

**Counselling**	24.5 [...] Prior to fertilisation of an ovum, extensive discussion and counseling is essential.	20.1 [...] You should ensure that appropriate counseling has been offered to the patient [...]	12. Counselling should be provided before, during and after treatment to those considering AHR treatment so that they are adequately informed of the risks involved, the potential benefits that may be obtained, and the possibility of success in their particular situation. Suitably qualified professionals should adequately convey the complex medical and scientific ramifications of different treatment approaches in verbal and written form.

**Embryo destruction**	24.5 [...] Any fertilised ovum must be used for normal implantation and must not be deliberately destroyed.	[*Repealed*]	10. Appropriate guidelines should be put in place by the regulatory body to govern the options available for excess frozen embryos. These would include [...] allowing them to perish.

**IVF indications**	24.5 Techniques such as IVF should only be used after thorough investigation has failed to reveal a treatable cause for the infertility.	20.1 Assisted human reproduction treatments, such as IVF, should only be used after thorough investigation has shown that no other treatment is likely to be effective.	17. Services should be available without discrimination on the grounds of gender, marital status or sexual orientation subject to consideration of the best interests of any children that may be born. Any relevant legislation on the provision of AHR services should reflect the general principles of the Equal Status Acts 2000-4 subject to the qualifications set out in section 4.8.

*Notes*: AHR = assisted human reproduction, IVF = *in vitro *fertilisation, fertilised ovum = embryo

^a ^A Guide to Ethical Conduct and Behaviour, 6^th ^Edition. Medical Council. Dublin 2004:1-44.

^b ^Guide to Professional Conduct and Ethics for Registered Medical Practitioners, 7^th ^Edition. Medical Council. Dublin 2009:1-61.

^c ^Report of the Commission on Assisted Reproduction (2005). Government of Ireland Publications: Dublin; 2005

#### 1. IVF indications

The council's 2004 guidelines indicate that IVF "should only be used after thorough investigation has failed to reveal a treatable cause of the infertility" [5]. In contrast, the 2009 guidelines describe IVF as a treatment which "should only be used after thorough investigation has shown that no other treatment is likely to be effective" [4]. While both versions encourage thorough diagnostic investigations, the 2004 guidelines suggest that IVF should be essentially limited to unexplained infertility. This position had changed by 2009, where IVF was regarded as best applied when "no other treatment is likely to be effective" in 2009.

This modification has potentially serious implications for patient choice regarding elective fertility treatment. For example, there are instances where a non-IVF treatment might be considered by a patient, but the alternate approach would be much less effective than IVF. No provision for this circumstance is made by Medical Council guidelines. Is it "appropriate practice" to provide IVF for an older patient who simply wishes to access IVF first, because it is the fertility treatment most likely to be effective [6]? As the Medical Council's guidelines are presently configured, a violation would seem likely in such a case. It is unclear why the Medical Council would seek this level of control over a realm of patient management not seen in other areas of Irish clinical medicine.

#### 2. Status and fate of human embryos

In 2004, Medical Council guidelines stipulated that fertilised ovum/embryos "must be used for normal implantation and must not be deliberately destroyed" [5]. Interestingly, this proscription against deliberate embryo destruction is missing from the Medical Council's 2009 guidelines [4]. Since the 2004 restriction is removed--but not replaced by any clarifying language--in the 2009 edition, the matter is left for individual practitioners to determine what should be done with non-transferred (surplus) embryos in Ireland.

Similarly, Medical Council guidelines specifically permitted the donation of unused, surplus embryos to other recipients in 2004, yet this "option to donate" was retracted in the council's 2009 guidelines [4]. Although there is mention of "donor programmes" which may be offered to patients, this is not described further. Similar wording used in the 2004 guidelines suggests that these donor programmes may in fact refer to sperm and ova donation, rather than embryos. Taken together, these two modifications significantly reconfigure public health policy on non-transferred embryos from IVF, such that embryo destruction is presently favoured over embryo donation. This is because the 2009 guidelines make it easier for Irish IVF clinics to destroy human embryos, while at the same time, make it harder to organise donation of surplus embryos to suitable recipients (*i.e*., other infertile couples) [7].

#### 3. Credentials, facilities and standards

In a major departure from the 2004 edition, the council's 2009 guidelines specify that ART should be provided only by "suitably qualified professionals, in appropriate facilities, and according to the international best practice" [4]. While this is a new emphasis on accreditation, the council did not articulate exactly what qualifications and/or experience is necessary to meet this standard either for Irish-trained IVF practitioners, or for those trained in other jurisdictions who may seek to practice here. At present, the Medical Council relies on the RCPI's Institute of Obstetricians & Gynaecologists to accredit general obstetricians & gynaecologists [8]. The Institute has not established how it will evaluate practice or accreditation issues that are limited to the sub-specialty area of fertility medicine/surgery [9]. Equally, the council does not explain how patients or doctors can know if an IVF clinic is operating in "appropriate facilities", what agency will inspect such premises, or how "appropriate" status should be determined and validated. The Medical Council did not sanction the current statutory oversight that already exists over Irish IVF units by the Irish Medicines Board [10], but proposed no specific alternative. The reference to "international best practice" is non-descriptive and unenforceable in the absence of additional refinement (*e.g*., who defines this standard?).

#### 4. Gamete donation

Current (2009) Medical Council guidelines state that donations which are part of a donor programme should be "altruistic and non-commercial" [4]. This aspect of the guidelines represents a new level of regulatory control over IVF in Ireland. A wide variation currently exists throughout the E.U. concerning the terms and conditions for gamete donors, depending on particular legal requirements in each jurisdiction [11-13]. Research elsewhere has found that nominal compensatory payment to gamete donors does not negate the altruistic motive for donation [14]. How this principle should be applied in Ireland remains unsettled [10] because the 2009 guidelines do not specify terms and conditions for gamete donor compensation in Ireland. The closely allied social issue of donor privacy (*i.e*. anonymous donation) [15-17] was not addressed in either the 2004 or the 2009 guidelines. While donor privacy was addressed in the 2005 CAHR report, these findings were not acknowledged by the Medical Council.

### Medical Council membership dynamics, 2004-2009

The Medical Practitioners Act 2007, [18] included significant changes for the Medical Council. Indeed, its statutory composition changed radically as a direct result of this legislation. The membership of the Medical Council was to shift away from its historic roster of physicians, to a new model where most of the council would not be medical professionals. Specifically, the 2007 Act required that of its 25 members, 9 appointees specifically must fulfil the criteria of "not and never [having] been a medical practitioner in the State or in another jurisdiction". Other parts of the 2007 Act brought the total number of "practicing physician" members on the council to six (of 25), resulting in an unusual "medical minority" [19] among Ireland's Medical Council. Accordingly, the council's 2004 and 2009 ethical guidelines were authorised and issued by Medical Councils with very different memberships.

### The CAHR: a largely discarded resource?

The Commission for Assisted Human Reproduction (CAHR) was established by government mandate in 2000; its comprehensive report was published in 2005. This cross-disciplinary panel of experts was charged with the task of creating a report on the "possible approaches to the regulation of all aspects of assisted human reproduction and the social, ethical and legal factors to be taken into account in determining public policy in the area". The result was a 151-page document covering potential ethical issues, sampling Irish public opinion, providing a list of recommendations covering all areas of assisted reproduction, exploring dissent of opinions among commission members, and comparing similar guidelines among other countries [2]. This report focused exclusively on assisted fertility policy in Ireland in concert with the medical registration work of the Medical Council; indeed, the commission's landmark report was issued the year following the council's 2004 guidelines.

While the CAHR report proposed 40 recommendations specifically addressing reproductive health policy in Ireland, the Medical Council applied only four of these to their 2009 guidelines. These four recommendations were in aspects of accreditation and gamete donation. Perhaps not surprisingly, the first recommendation set forth by the CAHR was that a "regulatory body should be established by an Act of the Oireachtas to regulate [assisted fertility] services in Ireland". Such a regulatory body would have responsibility for issuing licences for using ART in a clinical, laboratory, storage or research faculty. In Section 202 of the 2009 Medical Council guidelines, such language from the CAHR report appears to have helped shape the council's recommendation for "suitably qualified professionals, in appropriate facilities". Additionally, this section of the 2009 guidelines incorporated the CAHR recommendation concerning national statistics and longitudinal studies by proposing that the "follow-up of outcomes should be the norm". Likewise, the CAHR recommendation that "donors should not be paid nor should recipients be charged for donations" excluding costs directly related to ART services, also appears in the 2009 guidelines. No other components of the CAHR report were accessioned into the IMC's 2009 guidelines.

## Conclusions

Ireland, Poland and Romania are the only E.U. member states without formal regulation for IVF and related assisted fertility treatments [20]. As the number of IVF cycles initiated in Ireland continues to rise, the lack of comprehensive national legislation covering assisted reproductive treatments presents a health policy problem of significant dimensions. Even though no organised group has ever advocated the continued unregulated status of fertility treatments in Ireland, the entire topic has proven sufficiently contentious that no one has succeeded in overcoming the inertia of inaction. The resulting neglect of the issue by the Oireachtas has effectively handed the Medical Council a regulatory blank canvas, empowering the council to develop its own rules for IVF in Ireland. Our analysis concludes that the council has only partially actualised this opportunity at present.

How the Medical Council has chosen to manage regulation of the assisted fertility portfolio brings important consequences for Irish patients, their families, and their children-those present and those yet to be born. From a public policy perspective, the changes in Medical Council guidelines regarding when IVF should be used and what may happen to surplus embryos illustrate distinctly inconsistent regulatory themes. This is because the former case actively limits how physicians decide when an elective medical procedure should be performed, while the latter case passively enlarges the range of options for physicians regarding surplus IVF embryos. Against this uneven background, it would be helpful for the council to clarify their regulatory objectives with respect to how they think IVF should be provided in Ireland.

Our assessment of the evolution of Medical Council guidelines for assisted fertility treatments between 2004 and 2009 finds some areas of positive change for Ireland. These would include the council's support for improved accreditation, record keeping, and audit. However, other modifications in the ethical guidelines (if strictly interpreted) could restrict or impair access to IVF at a time where demand for such treatment is increasing in Ireland. That the council distanced itself from the key dilemma of what to do with surplus (non-transferred) frozen embryos between 2004 and 2009 was an unexpected finding of this analysis. While the 2004 guidelines stated that embryos must not be deliberately destroyed, those same guidelines did recognise the option of potential donation to other individuals [5]. Curiously, the 2009 guidelines are now silent as to what can be done with excess embryos in Ireland [4]. Whatever protections these embryos may have had in 2004, the Medical Council considerably weakened such protection by 2009. While the sponsors of the Medical Practitioners Act, 2007 may not have intended embryos from IVF to be more vulnerable after the required membership reorganisation at the Medical Council, current (2009) practice guidelines have achieved this very effect. It is also impossible to know if this erosion of protection for IVF embryos would have happened if the Medical Council had retained its medical majority composition, but this seems unlikely based on its historically "pro life" ethical emphasis.

Recent research has underscored the complexities associated with donor compensation and privacy [21,22], as well as embryo disposition [10,23] and abandonment [24] in Ireland. It is disappointing that none of these important issues were explored in the 2009 guidelines. We agree with the CAHR report that the ultimate responsibility for regulation of assisted fertility services in Ireland rests with the Oireachtas [25,26]. But by now, the Medical Council may need to confront the uncomfortable possibility that the Oireachtas might never achieve this. Indeed even if it does enact such legislation, many years could pass in the process. In the meantime, we continue our endorsement of the Medical Council's statement (appearing in the *Introduction *to their current 2009 guidelines) that comprehensive guidelines must be developed to address the issues highlighted here.

## Competing interests

The Sims Institute is an independent, non-government clinical & research organisation; none of the authors report any conflict of interest.

## Authors' contributions

DJW was lead investigator and principal consultant, MLM was medical student, and ESS developed the project and supervised the overall research. All authors read and approved the final manuscript.
